# Differential Induction of Isolated Lymphoid Follicles in the Gut by 18β-Glycyrrhetinic Acid

**DOI:** 10.1371/journal.pone.0100878

**Published:** 2014-07-03

**Authors:** Jay M. Hendricks, Diana C. Lowe, Michele E. Hardy

**Affiliations:** 1 Department of Veterinary Microbiology and Pathology, College of Veterinary Medicine, Washington State University, Pullman, Washington, United States of America; 2 Immunology and Infectious Diseases, Montana State University, Bozeman, Montana, United States of America; Toho University School of Medicine, Japan

## Abstract

18β-glycyrrhetinic acid (GRA) is a pharmacologically active component of licorice root with documented immunomodulatory properties. We reported that GRA administered orally to mice induces B cell recruitment to isolated lymphoid follicles (ILF) in the small intestine and shortens the duration of rotavirus antigen shedding. ILF are dynamic lymphoid tissues in the gut acquired post-natally upon colonization with commensal bacteria and mature through B cell recruitment to the follicles, resulting in up-regulation of IgA synthesis in response to changes in the composition of microbiota. In this study, we investigated potential mechanisms by which GRA induces ILF maturation in the ileum and the colon using mice depleted of enteric bacteria and a select group of mice genetically deficient in pattern recognition receptors. The data show GRA was unable to induce ILF maturation in ileums of mice devoid of commensal bacteria, MyD88^−/−^ or NOD2^−/−^ mice, but differentially induced ILF in colons. Increased expression of chemokine and chemokine receptor genes that modulate B and T cell recruitment to the mucosa were in part dependent on NOD2, TLR, and signaling adaptor protein MyD88. Together the results suggest GRA induces ILF through cooperative signals provided by bacterial ligands under normal conditions to induce B cell recruitment to ILF to the gut, but that the relative contribution of these signals differ between ileum and colon.

## Introduction

18β-glcyrrhetinic acid (GRA) is the aglycone metabolite of glycyrrhizin, a pharmacologically active component of licorice root. Glycyrrhizin is rapidly hydrolyzed in the gut by bacterial glucuronidases to GRA [1], and data continue to accumulate that suggest this compound modulates several parameters of the immune response to both infectious and non-infectious diseases. Antibacterial, antiviral, anti-allergic, anti-inflammatory, and some pro-inflammatory properties have been described *in vitro* in various cell types and lines, and *in vivo* in mouse models (reviewed in [2]). Of note, glycyrrhizin has been in use for many years in Japan as an intravenous treatment for chronic hepatitis [3], [4]. Activation or inhibition of transcription factors, phosphatases, kinases and nitric oxide synthase all have been reported [5]–[11]. How all of these key observations coalesce into what can be considered typical responses to GRA is important to understand, and is complicated by experiments performed in different cell lines or animal models that utilize different stimuli and routes of administration. Regardless, there are sufficient data to support *in vivo* biological activity of GRA, making this an attractive compound to investigate for its ability to induce or modulate beneficial immune responses. Importantly, experimental evidence for activity in the intestine following oral delivery has been documented [5], [12]. There are significant advantages to orally administered compounds in the context of development of adjuvants and immunomodulatory therapeutics, and GRA has potential to function in this capacity.

We reported that GRA administered orally to mice induces B cell recruitment to isolated lymphoid follicles (ILF) in the gut and does so in the absence of external antigenic stimulus [12]. ILF are dynamic B cell-rich lymphoid tissues that, in contrast to secondary lymphoid tissues including Peyer’s Patches (PP) and lymph nodes, develop post-natally upon acquisition of commensal bacteria (reviewed in [13]). ILF are present in both the small intestine and colon, and numbers of ILF increase in the ileum extending in to the colon as the concentration of bacteria increases distally [14], [15]. ILF consist of a spectrum of structures with size and cellular composition that is characteristic of maturation status [16]. The number of ILF in the gut is invariant, but those present are morphologically dynamic, and thus have been collectively termed solitary isolated lymphoid tissue (SILT) [16]. SILT initially derive from cryptopatches, precursor structures located at the base of the crypts that are formed independently of bacterial colonization [16], [17]. Immature ILF are induced by initial acquisition of enteric microbiota and consist of few B220^+^ B cells framed by CD11c^+^ dendritic cells (DC), and few CD3^+^ T cells. Upon induction with appropriate signals, B cells are recruited to ILF to form germinal centers that displace the lamina propria, and by mechanisms not entirely understood, develop a follicle-associated epithelium containing M cells similar to PP [18]. ILF serve as inductive sites for IgA synthesis, and their maturation to large B cell follicles occurs at least in part in response to changes in the composition of bacterial populations and some dietary ligands [14], [19]–[21]. ILF thus play a significant role in maintenance of intestinal inflammatory homeostasis by regulating mucosal IgA synthesis to control potentially damaging fluctuations in the microbiome.

Signals that stimulate induction and maturation of ILF have been revealed through studies in knockout mice as well as germ-free, and differentially colonized mice [13], [14], [19], [22]–[26]. Chemokines and chemokine receptors associated with B cell recruitment including CXCL13, CXCR5, CCL20 and CCR6 are important for ILF maturation in the ileum, as are receptor activator of NFκB ligand RANKL and β-defensin 3 [19], [24], [25], [27]. Importantly, and consistent with a primary role of bacterial flora in ILF development in the small intestine, ILF are not present in ileums of mice deficient in intracellular pattern recognition receptor NOD1 [22]. Notably, ILF appear altered either in total numbers of immature and mature ILF, or as proportional changes between the two in mice deficient in other pattern recognition receptors or signaling molecules including TLR2/4, NOD2, MyD88 and Trif, and these proportions vary between ileum and colon [22]. For example, although ILF are not present in ileums of NOD1^−/−^ mice, mature ILF are hypertrophic in the colons [22], illustrating the involvement of multiple signaling molecules with prominent roles in the innate immune response that contribute to ILF maturation.

We reported that GRA administered orally induces ILF maturation in the ileum and in addition, shortens the duration of rotavirus antigen shedding in the mouse model [12]. The experiments described in the current study investigated whether GRA influences ILF maturation in the colon, and sought to understand mechanisms by which GRA induces B cell recruitment to ILF.

## Materials and Methods

### GRA

18β-glycyrrhetinic acid was purchased from Sigma-Aldrich. Stock solutions were prepared to a concentration of 100 mg/mL in DMSO and diluted to working concentration in calcium and magnesium-free phosphate buffered saline (PBS). Endotoxin levels were measured by Limulus Amoebocyte Lysate Assay (Associates of Cape Cod, Inc), and the final amount of endotoxin present in doses delivered to mice was <0.025 EU.

### Mice and GRA treatments

#### Ethics statement

All animal experiments were performed according to the NIH Guidelines for Care and Use of Animals with protocol approval from the Montana State University (Protocol number 2011-44) and Washington State University (Protocol number 00453-004) Institutional Animal Care and Use Committees. Male C57Bl/6, C3H/HeJ, NOD2^−/−^, and MyD88^−/−^ mice were obtained from Jackson Laboratories. Animals were co-housed according to treatment group under SPF conditions, with ad libitum access to food and water. All experiments were performed when animals were eight weeks of age and all groups contained five mice/group. GRA treatments and rotavirus infections were performed as previously described [12]. Briefly, mice were administered 50 mg/kg of GRA by oral gavage on day one and then a second time on day three. No adverse effects were observed in GRA treated mice. In experiments where groups included rotavirus infected mice, 10^5^ shedding dose 50 (SD_50_) of murine rotavirus strain EW was administered by oral gavage on day two. EW does not cause diarrhea or other illness in mice >15 days of age, and infection is measured by antigen shedding in feces. Mice were sacrificed on day eleven by overdose CO_2_ inhalation.

### Histology

Tissue sections from ileum and colon were harvested as described previously [12]. Sections were rinsed with PBS to eliminate intestinal contents, and then infused with OCT. Tissue sections were coiled into a cryomold with the proximal end at the center, covered with OCT, and then snap frozen in liquid nitrogen. Five µM thick sections were mounted on Superfrost slides (Fisher), and fixed with 75% acetone/25% ethanol for five minutes, air dried and then stained with antibodies to B220 (A488), CD11c (PE), CD35 (PE) or CD3e (PE), all from eBiosciences.

### Ablation of intestinal bacteria

Bacteria were ablated from the intestines of C57Bl/6 mice as previously described [28]. Mice were administered an antibiotic cocktail containing 10 mg each of ampicillin, vancomycin, metranidozol and neomycin by oral gavage daily for five days, followed by five days of continuous administration in the drinking water. The same cocktail was present continuously in the drinking water at a concentration of one gram/L through the course of the experiment. No adverse affects of daily oral gavage or continuous antibiotic treatment were observed. The absence of bacteria was confirmed by plating 10% fecal homogenates on bovine heart infusion agar containing 10% FBS.

### Transfections and reporter assays

HEK293 cells were cultured in DMEM containing 10% FBS. Cells were co-transfected with NOD2 expression plasmid pUNO-mNOD2 (InVivoGen), pNFκB-luc-*cis* reporter and phRL renilla luciferase reporter plasmids (Promega). Twenty-four hours post-transfection, cells were treated with GRA and reporter gene expression was measured with the Dual-Glo Luciferase Assay (Promega). Data are presented as percent response relative to the NOD2 ligand muramyl dipeptide control set to 100%. Data were analyzed by unpaired *t* test. Error bars represent standard error of the mean of three separate experiments; p<0.05.

### Quantitative PCR and cytokine arrays

Gene expression in intestinal tissue induced by GRA was measured by RT-qPCR and custom gene arrays as previously described [12]. Ten hours post-GRA administration, sections of ileum and colon were collected and stored in RNAlater (Qiagen). RNA was extracted with the RNeasy system (Qiagen) and quantified with a Nanodrop 1000 (Fisher Scientific). Cytokine transcripts were measured with the SABiosciences Custom Mouse RT^2^ Profiler™. Custom arrays included Cxcr5, Ccl19, Ccl21b, Cxcl13, Lta, Ltb, Ccr6, Ccr7, Ccr9, Il10, Il6, Ccl20, and Ccl25. One µg of RNA was reverse transcribed with RT^2^ First Strand kit (SABiosciences) and PCR reactions were performed on an Eppendorf Realplex 4s under reaction conditions of 95°C for 10 minutes, followed by 40 cycles of 95°C for 15 seconds, and 60°C for one minute. Data are from a minimum of three mice per group and are expressed as fold-change over vehicle-treated animals. Fold-changes >2 were considered significant.

## Results

### ILF are not induced by GRA in ileums of antibiotic treated mice, but are induced in colons

**Figure 1:**
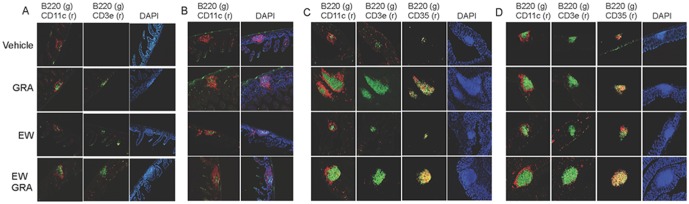
GRA induces ILF in the colon, but not in the ileum of antibiotic treated mice. Intestinal bacteria were ablated by daily oral gavage with an antibiotic cocktail daily for five days and then with the same cocktail in the drinking water for an additional five days prior to administration of GRA. The same cocktail was maintained in the drinking water through the course of the experiment. Animals infected with rotavirus strain EW were given 10^5^ SD_50_ of virus inoculum by oral gavage. A) ileum; normal flora, B) ileum; antibiotic treated, C) colon; normal flora and D) colon; antibiotic treated. Tissue sections in (A) were stained for B cells (B220), T cells (CD3e) and dendritic cells (CD11c). Panel (B) does not include CD3e staining because T cells were not located in the absence of B220^+^ aggregate staining. Sections in C) and D) were strained for B cells, dendritic cells, T cells, and follicular dendritic cells (CD35). The labels on the left indicating treatments apply across rows for A–D. Magnification 10X.

Toward a goal of understanding mechanisms by which GRA induces ILF by defining signaling pathways involved and the initial role of commensal bacterial ligands, intestinal bacteria were ablated with an antibiotic cocktail, and then administered GRA orally on day one, and then a second time two days later. Tissue sections from the distal ileum and colon were harvested on day eleven. As reported previously, histological staining for B cells (B220), DC (CD11c), and T cells (CD3) in the ileum revealed that GRA induced B220^+^ cell recruitment to lamina propria ILF (Figure 1A). However, GRA did not induce ILF in the ileum in antibiotic treated mice, as indicated by the lack of B cell aggregates. (Figure 1B). Colonic ILF maturation was induced by GRA in mice with normal flora (Figure 1C), and in contrast to results in the ileum, induced significant B220^+^ B cell recruitment to ILF in the absence of bacteria (Figure 1D). Small ILF are present in colons of vehicle treated mice as a result of original bacterial colonization. CD35^+^ follicular dendritic cells indicative of more mature ILF also were observed, consistent with previous observations that colonic ILF are inherently larger in the steady state than ileal ILF. We further note that colonic ILF appear somewhat larger in antibiotic treated mice than in normal mice, but the basis for this difference is not clear.

We reported a notable increase in B220^+^ cell clusters in the ileums of rotavirus infected C57Bl/6 mice nine days post-infection, and sizes of B220^+^ aggregates were enhanced by GRA treatment [12]. In rotavirus infected mice absent enteric bacteria, virus infection alone induced a noticeable, but small increase in B220^+^ cell aggregates in both ileum and colon (Figure 1B&D). Enhancement of B cell recruitment in GRA treated, rotavirus infected mice was not apparent when compared to GRA treated, uninfected mice in either tissue.

Taken together, these data are consistent with those reported by others that describe differential roles of commensal bacteria in modulating ILF development between the ileum and colon [19], [22], [26]. The data suggest that in the ileum, GRA does not stimulate signaling pathways that can bypass the need for bacterial ligands to induce B cell recruitment to ILF. In contrast, GRA-mediated B cell recruitment to colonic ILF is largely independent of substantial bacterial ligand-responsive signaling. This conclusion is drawn with the caveat that there may be residual bacteria not ablated by the antibiotic treatment regimen that could contribute to TLR mediated signaling that would drive B cell recruitment.

### GRA induces ILF in ileums and colons of C3H/HeJ mice

**Figure 2:**
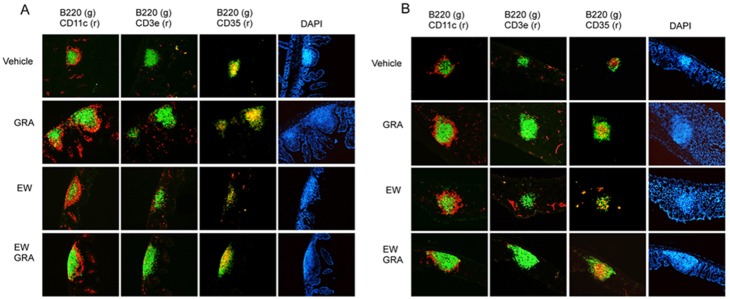
GRA induces ILF maturation in ileum and colon of C3H/HeJ mice. Mice were administered GRA according the schedule described in the materials and methods. Animals infected with rotavirus strain EW were given 10^5^ SD_50_ of virus inoculum by oral gavage. Tissue sections were stained for B cells (B220), dendritic cells (CD11c), T cells (CD3), and follicular dendritic cells (CD35). A) ileum and B) colon. Magnification 10X.

GRA did not induce ILF in the ileums of mice in the absence of bacteria, but induced significant B cell recruitment to colonic ILF. To further investigate a role for TLR signaling in GRA-mediated ILF maturation, the ability of GRA to induce ILF maturation in C3H/HeJ mice was tested. C3H/HeJ mice harbor a spontaneous mutation that renders animals defective in TLR4 responses [29]. In these mice, GRA induced B cell recruitment to both the ileum and colon, and numerous CD35^+^ cells were observed (Figure 2). ILF in rotavirus infected mice were not appreciably different than uninfected mice in either ileum or colon, and were not enhanced beyond those induced by GRA alone. The ability of GRA to induce ILF maturation in ileum and colon of C3H/HeJ mice suggests robust TLR4 responses do not play a significant role in GRA-mediated B cell recruitment in either tissue, but these results do not rule out a role for other TLR. In addition, these observations further reinforce that reactivity to any incidental introduction of endotoxin is not an explanation for induction of ILF maturation by GRA.

### MyD88 is required for GRA induced B cell recruitment to ILF in ileum and colon

**Figure 3:**
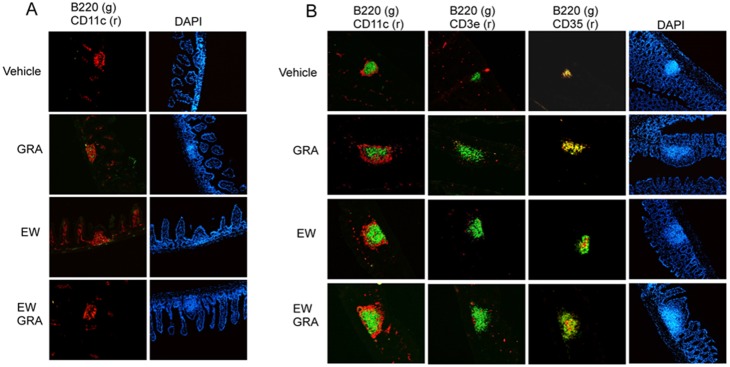
GRA does not induce B cell recruitment to ILF in MyD88^−/−^ mice. Mice were administered GRA according the schedule described in the materials and methods. Animals infected with rotavirus strain EW were given 10^5^ SD_50_ of virus inoculum by oral gavage. Tissue sections from the ileum (A) were stained for B cells (B220) and dendritic cells (CD11c). Panels for T cell (CD3a) and CD35 staining were negative and so not included in the figure. Tissue sections from the colon (B) were stained for B cells, T cells, and follicular dendritic cells. Magnification 10X.

The signaling adaptor protein MyD88 is required to propagate most TLR responses, as well as for signaling through the IL-1 receptor [30]. Immature ILF are present in MyD88^−/−^ mice, but MyD88 is required for authentic ILF maturation in both ileum and colon [26]. Consistent with this, B220^+^ cell aggregates indicative of ILF were virtually absent in the ileums of MyD88^−/−^ mice and were not induced by GRA treatment or by rotavirus infection (Figure 3). Small B220^+^ cell aggregates were present in the colons of vehicle treated MyD88^−/−^ mice, but there were not appreciable increases in size upon administration of GRA. Some CD35^+^ cell staining associated with B cell aggregates was observed in colon tissue under both conditions, but assessment of additional cellular markers is required to determine whether GRA induces significant maturation in the absence of enhanced B cell recruitment. These data suggest MyD88 is indispensable for GRA-mediated B cell recruitment to ILF in both tissues, suggesting a role for TLR signaling, without excluding a potential role for MyD88 signaling through the IL-1R.

### ILF are not induced by GRA in NOD2^−/−^ mice

**Figure 4:**
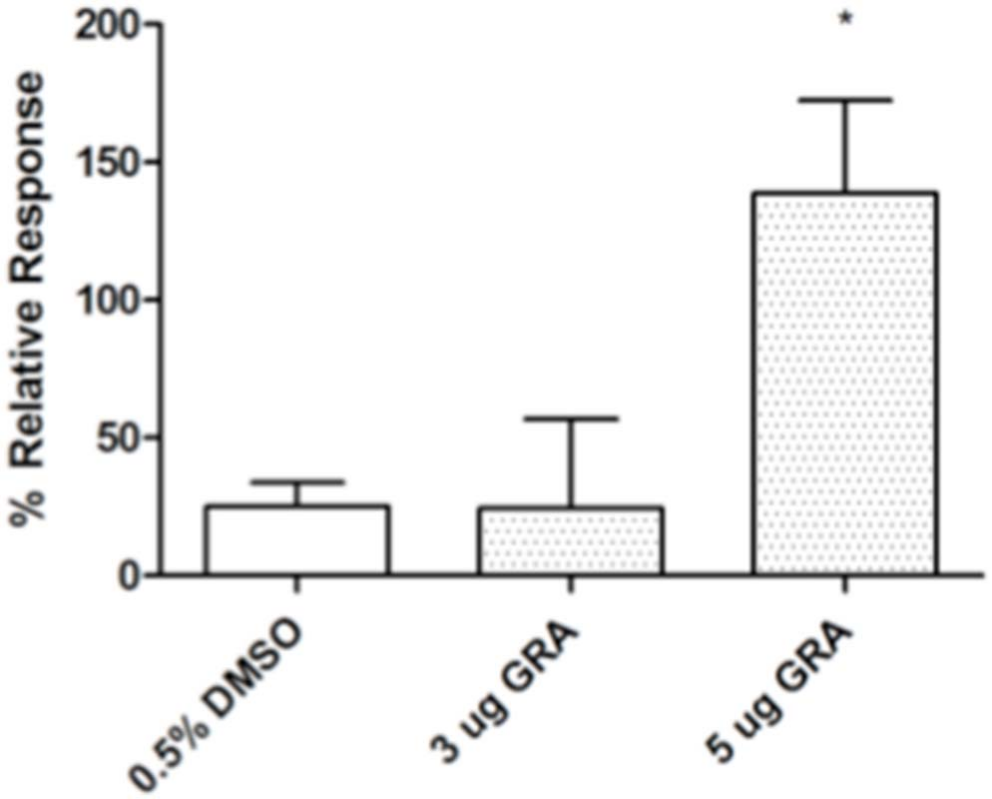
GRA activates NOD2-mediated reporter gene expression in vitro. Cells were co-transfected with NOD2 expression plasmid pUNO-mNOD2 (InVivoGen), NFkB-luc-*cis* reporter and phRL renilla luciferase reporter plasmids. Twenty-four hours post-transfection, cells were treated with GRA and reporter gene expression was measured with Dual-Glo Luciferase Assay (Promega). Data are expressed as percent response relative to the NOD2 ligand muramyl dipeptide control set to 100%. Error bars represent standard error of the mean of three separate experiments. P<0.05.

**Figure 5:**
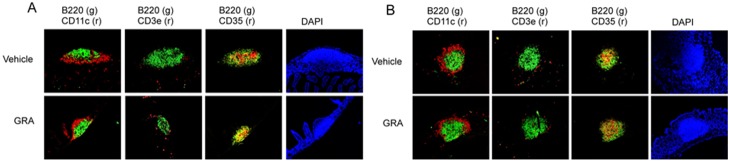
GRA does not induce B cell recruitment to ILF in NOD2^−/−^ mice. Mice were administered GRA according the schedule described in the materials and methods. Tissue sections were stained for B cells (B220), dendritic cells (CD11c), T cells (CD3), and follicular dendritic cells (CD35). A) ileum and B) colon. Magnification 10X.

Pattern recognition receptors NOD1 and NOD2, in addition to TLR, play critical roles in driving innate immune responses in the gut, in part through interactions with membrane components of commensal bacteria [31], [32]. NOD1 is key to ILF development in the small intestine because ILF are not induced in the ileums of NOD1^−/−^ mice [22]. NOD1 also functions in ILF development in the large intestine because mature ILF are hypertrophic. Induction of ILF in the ileum or in the colon is not substantially altered in NOD2^−/−^ mice, but progression to mature ILF in both tissues is incomplete [22], [26]. Given the role for both TLR and NOD receptors in ILF maturation, we tested reactivity of GRA with selected receptors in reporter systems *in vitro*. GRA did not activate TLR reporter gene expression in the THP-1 Blue NFκB reporter activation system that responds to TLR2, TLR1/2, TLR2/6, TLR4, TLR5 and TLR8 ligands (InVivoGen), nor did it activate NOD1-mediated reporter gene expression in transient transfections (data not shown). However, GRA did activate NOD2-mediated gene expression (Figure 4). Based on these data, the ability of GRA to induce B cell recruitment to ILF in NOD2^−/−^ mice was tested. Few B cell aggregates also staining positive for CD35 were observed in the ileums and colons of both vehicle treated and GRA treated mice (Figure 5). There were no appreciable differences in size between treated and vehicle treated mice. Similar to MyD88^−/−^ mice, delineation of additional markers of maturation status are required to determine whether GRA can induce maturation independently of enhanced B cell recruitment. Together, the data suggest that NOD2 activation as well as TLR signaling, plays a role in GRA-mediated ILF maturation.

### GRA-induced chemokine and chemokine receptor gene expression in ileum and colon of mouse models

We previously reported that GRA induced a specific pattern of chemokine and chemokine receptor gene expression in the ileum that was predictive of B cell recruitment to the gut [12]. A similar analysis was performed on RNA collected from ileum and colon 10 hours following oral administration of GRA in each of the mouse models studied here. The gene expression pattern in ileums of antibiotic treated mice, C3H/HeJ, and MyD88^−/−^ mice was similar to that of wild type mice (Table 1). In contrast, this pattern of expression was not observed in ileum from NOD2^−/−^ mice. Opposing results were observed in colon tissue. Increased expression of selected genes was not observed in colons of antibiotic treated, C3H/HeJ, or MyD88^−/−^ mice, but expression in NOD2^−/−^ mice was similar to wildtype mice. These data again suggest a cooperative role for NOD2 and TLR signaling in the mechanism of GRA-mediated ILF maturation because GRA induced gene chemokine and chemokine receptor gene expression in the absence of TLR signaling, but was unable to induce B cell recruitment to ILF. The presence of NOD2 appears to be required for gene expression in the ileum and also required cooperatively for ILF maturation. In contrast, TLR signaling appears required for GRA-induced gene expression and for robust B cell recruitment to ILF in the colon. The latter conclusion is based primarily on observations in the MyD88^−/−^ and NOD2^−/−^ mice, since it is possible that residual bacteria following antibiotic ablation that are not readily cultivatable may provide sufficient signaling through TLR.

**Table 1:** GRA-induced gene expression in ileum and colon of select mouse models.

	WT		Antibiotic Treated		C3H/HeJ		MyD88^−/−^		NOD2^−/−^	
	Ileum	Colon	Ileum	Colon	Ileum	Colon	Ileum	Colon	Ileum	Colon
Cxcr5	**15.74**	**10.44**	**15.98**	1.04	**3.82**	1.83	**6.71**	−1.55	−2.61	**10.08**
Ccl19	**4.26**	**4.68**	**5.57**	**2.16**	**3.01**	1.18	**7.80**	1.17	−1.51	**3.58**
Ccl21b	1.49	1.09	1.96	1.06	1.52	1.21	1.42	−1.13	1.58	1.23
Cxcl13	**3.08**	**2.73**	1.98	1.21	**2.79**	1.65	**3.49**	1.31	−1.35	**2.36**
Lta	**4.75**	**2.64**	**4.78**	1.28	0.99	1.43	**2.33**	−1.23	−1.75	**2.64**
Ltb	**4.49**	**5.33**	**3.17**	1.09	1.96	1.49	**3.10**	−1.22	−1.24	**3.42**
Ccr6	**7.91**	**6.50**	**5.69**	1.41	**2.12**	1.62	**5.63**	−1.00	−2.22	**6.26**
Ccr7	**4.87**	**7.72**	**4.59**	1.19	**2.54**	1.51	**5.86**	−1.30	−1.73	**3.81**
Ccr9	1.41	1.70	−1.61	1.02	1.31	1.44	1.26	1.02	−1.19	1.05
IL10	**2.50**	**2.14**	1.09	−1.09	1.83	1.12	1.25	−1.04	−1.05	1.29
IL6	**2.40**	1.32	1.18	−1.02	1.85	1.27	1.24	−1.81	1.28	1.00
Ccl20	ND	1.93	1.24	1.28	**2.50**	1.76	1.13	1.42	−1.85	**4.58**
Ccl25	ND	1.03	−1.52	1.21	1.10	1.12	−1.11	−1.08	1.41	1.16

Data presented are fold-increase in GRA-treated animals relative to vehicle treated controls, and are representative from multiple experiments. Bold text designates values with increases above arbitrary 2-fold cut-off.

## Discussion

The contributions of dietary ligands to modulation of dynamic lymphoid tissue in the gut are just beginning to be explored. Mice fed diets free of defined phytochemicals have reduced numbers of both cryptopatches and ILF, implicating a key role for nutrients in maintaining (or disrupting) intestinal inflammatory homeostasis [20], [33]. We have explored the mechanisms by which dietary ligand GRA induces B cell recruitment to ILF. Our initial studies on GRA focused on the small intestine, yet it is clear that many of the molecular signals required for ILF development in the ileum are dispensable in the colon. Given the tissue-specific signaling requirements, we extended our studies to include analysis of GRA-mediated B cell recruitment to ILF in the colon. We tested the ability of GRA to induce ILF in mice depleted of enteric bacteria, and in mice with genetic deficiencies in components of the innate immune response to define potential mechanisms of action.

Collective interpretation of data regarding GRA-induced gene expression and B cell recruitment to ILF leads us to propose that a mechanistic role for both TLR signaling and NOD2 signaling differs between small intestine and colon. GRA-induced chemokine and chemokine receptor gene expression in ileums of antibiotic treated mice and MyD88^−/−^ mice suggest TLR signaling is not required for up-regulation of gene expression, but is required for B cell recruitment to ILF because ILF were not observed. Activation of NOD2 also is implicated because GRA was unable to induce chemokine and receptor gene expression in ileums of NOD2^−/−^ mice, nor were B cells recruited to ILF even though these mice are normally colonized. Cellular responses to NOD2 activation are propagated through activation of NFκB [34], [35]. We and others have reported that GRA activates NFκB both *in vitro* and *in vivo* [36] [5] supporting sthe suggestion that GRA may interact directly or indirectly with this receptor. If so, GRA thus represents a unique NOD2-activating dietary ligand.

In contrast to the ileum, GRA did not induce chemokine and chemokine receptor gene expression in colons of antibiotic treated or MyD88^−/−^ mice, whereas B cell recruitment was induced in the former, but not the latter. The lack of induced gene expression and ILF maturation in colons of MyD88^−/−^ mice points to a role for TLR signaling, or alternatively, signaling through the IL1R. In contrast, although gene expression was up-regulated in colons of NOD2^−/−^ mice, robust B cell recruitment to ILF was not observed. The difference in gene expression in ileum and colon compared to the other mouse models is not clear, but in the NOD2^−/−^ mice, induction may be driven more by signaling through TLR in the presence of expanded bacterial colonization in these mice, although such differences recently have been called to question [37]. In the context of a mechanism for the ability of GRA to induce B cell recruitment, our data significantly implicate both TLR and NOD2 signaling similar to previous studies suggesting synergistic roles for NOD2 and TLR in innate immune responses in the gut [38], but the precise mechanisms and cell types acted on remain to be determined.

ILF function as inductive sites for IgA synthesis in part in response to changes in gut microbiota [13]. While the ability of GRA to induce B cell recruitment to ILF is clear, the mechanism by which recruitment takes place is not known. We have shown that GRA induces a pattern of gene expression in ileums of C57Bl/6 mice, and now colons, within ten hours following oral administration. This pattern is predictive of B cell recruitment, namely CXCR5, CXCL13, CCR6, CCL19/20 and lymphotoxin a and b, all known to play key roles [13]. Consistent with differences between ILF development in ileum and colon, the CXCR5-CXCL13 and CCR6-CCL20 axes are indispensable for authentic ILF maturation in the ileum, but not in the colon [19], [22], [26]. GRA may directly induce B cell recruitment to ILF by inducing expression of these genes, posing the question of what cell types are responsive. In this context, it is tempting to speculate that DC are a prominent source of chemotactic cytokines. GRA has been shown to promote DC maturation *in vitro* [39], [40], but studies to support DC maturation from antigen processing to antigen presenting functions *in vivo* following exposure by the oral route have not been reported.

Evidence for NOD2 activation both *in vitro* and *in vivo* brings forth the intriguing possibility that GRA induces secretion of antimicrobial peptides that result in alterations in the composition of the microbiota. NOD2 is highly expressed in Paneth cells localized in the small intestinal crypts and play a prominent role in controlling both commensal and pathogenic bacteria through up-regulated expression of antimicrobial peptides [41]. Whether antimicrobial peptide activity is increased by GRA remains to be determined.

Glycyrrhizin and GRA are inhibitors of 11β-hydroxsteroid dehydrogenase 1 (11βHSD1) which catalyzes conversion of cortisone to cortisol, resulting in suppression of inflammation [2]. Recent data show T cell responses are enhanced following vaccination with an antigen targeted to DC when a TLR ligand was administered in coordination with pharmacological inhibition of 11βHSD [42]. Thus it is worthwhile to speculate that interactions between 11βHSD and GRA in coordination with TLR and NOD2 signaling are contributing to ILF maturation. In addition, the enhanced T cell response observed when 11βHSD1 was inhibited implicates a potential of GRA to function as an adjuvant, and data exist to support this capability [9].

Orally delivered GRA shortens the duration of rotavirus antigen shedding in the adult mouse model [12]. CD8^+^ T cells are significantly increased in PP in GRA treated, rotavirus infected C57Bl/6 mice and endpoint anti-rotavirus serum antibody titers were slightly increased. In the previous study, an apparent increase in B220^+^ cell aggregates in rotavirus infected mice that were not treated with GRA was noted, and the sizes of these aggregates were enhanced in GRA treated, infected mice. Rotavirus infection in the current study did not result in B cell recruitment either the presence or absence of GRA in any of the mouse models, suggesting increases in B cell recruitment to ILF as a result of rotavirus infection requires all of the components tested here, including pattern recognition receptors and signaling adaptor MyD88. Studies to determine mechanisms by which GRA reduces the duration of rotavirus antigen shedding, including T cell proliferation and generation of rotavirus-specific IgA are ongoing.

## References

[1] Hattori M, Sakamoto T, Kobashi K, Namba T (1983) Metabolism of glycyrrhizin by human intestinal flora. Planta Med 48: 38–42. 6611743 10.1055/s-2007-969875

[2] Asl MN, Hosseinzadeh H (2008) Review of Pharmacological Effects of Glycyrrhiza sp. and its Bioactive Compounds. Phytother Res 22: 709–724 10.1002/ptr.2362 18446848 PMC7167813

[3] van Rossum TG, Vulto AG, de Man RA, Brouwer JT, Schalm SW (1998) Review article: glycyrrhizin as a potential treatment for chronic hepatitis C. Alimentary Pharmacology & Therapeutics. 12: 199–205. 10.1046/j.1365-2036.1998.00309.x 9570253

[4] Ikeda K, Arase Y, Kobayashi M, Saitoh S, Someya T, et al (2006) A Long-Term Glycyrrhizin Injection Therapy Reduces Hepatocellular Carcinogenesis Rate in Patients with Interferon-Resistant Active Chronic Hepatitis C: A Cohort Study of 1249 Patients. Dig Dis Sci 51: 603–609 10.1007/s10620-006-3177-0 16614974

[5] Ukil A, Biswas A, Das T, Das PK (2005) 18 Beta-glycyrrhetinic acid triggers curative Th1 response and nitric oxide up-regulation in experimental visceral leishmaniasis associated with the activation of NF-kappa B. J Immunol. 175: 1161–1169. 10.4049/jimmunol.175.2.1161 16002718

[6] Ukil A, Kar S, Srivastav S, Ghosh K, Das PK (2011) Curative effect of 18β-glycyrrhetinic acid in experimental visceral leishmaniasis depends on phosphatase-dependent modulation of cellular MAP kinases. PLoS ONE 6: e29062 10.1371/journal.pone.0029062 22194991 PMC3237588

[7] Kao T-C, Shyu M-H, Yen G-C (2010) Glycyrrhizic Acid and 18β-Glycyrrhetinic Acid Inhibit Inflammation via PI3K/Akt/GSK3β Signaling and Glucocorticoid Receptor Activation. J Agric Food Chem 58: 8623–8629 10.1021/jf101841r 20681651

[8] Wang C-Y, Kao T-C, Lo W-H, Yen G-C (2011) Glycyrrhizic Acid and 18β-Glycyrrhetinic Acid Modulate Lipopolysaccharide-Induced Inflammatory Response by Suppression of NF-κB through PI3K p110δ and p110γ Inhibitions. J Agric Food Chem 59: 7726–7733 10.1021/jf2013265 21644799

[9] Kim J, Joo I, Kim H, Han Y (2013) Phytomedicine. European Journal of Integrative Medicine 20: 951–955 10.1016/j.phymed.2013.04.008

[10] Fiore C, Eisenhut M, Krausse R, Ragazzi E, Pellati D, et al (2008) Antiviral effects ofGlycyrrhiza species. Phytother Res 22: 141–148 10.1002/ptr.2295 17886224 PMC7167979

[11] Chang Y-L, Chen C-L, Kuo C-L, Chen B-C, You J-S (2010) Glycyrrhetinic acid inhibits ICAM-1 expression via blocking JNK and NF-κB pathways in TNF-α-activated endothelial cells. Acta Pharmacol Sin 31: 546–553 10.1038/aps.2010.34 20418897 PMC4002749

[12] Hendricks JM, Hoffman C, Pascual DW, Hardy ME (2012) 18β-Glycyrrhetinic Acid Delivered Orally Induces Isolated Lymphoid Follicle Maturation at the Intestinal Mucosa and Attenuates Rotavirus Shedding. PLoS ONE 7: e49491 10.1371/journal.pone.0049491.t001 23152913 PMC3496704

[13] Knoop KA, Newberry RD (2012) Isolated Lymphoid Follicles are Dynamic Reservoirs for the Induction of Intestinal IgA. Front Immunol 3 10.3389/fimmu.2012.00084 PMC3343265 22566964

[14] Lorenz RG, Chaplin DD, McDonald KG, McDonough JS, Newberry RD (2003) Isolated lymphoid follicle formation is inducible and dependent upon lymphotoxin-sufficient B lymphocytes, lymphotoxin beta receptor, and TNF receptor I function. J Immunol 170: 5475–5482. 12759424 10.4049/jimmunol.170.11.5475

[15] Hamada H, Hiroi T, Nishiyama Y, Takahashi H, Masunaga Y, et al (2002) Identification of multiple isolated lymphoid follicles on the antimesenteric wall of the mouse small intestine. J Immunol 168: 57–64. 11751946 10.4049/jimmunol.168.1.57

[16] Pabst O, Herbrand H, Worbs T, Friedrichsen M, Yan S, et al (2005) Cryptopatches and isolated lymphoid follicles: dynamic lymphoid tissues dispensable for the generation of intraepithelial lymphocytes. Eur J Immunol 35: 98–107 10.1002/eji.200425432 15580658

[17] Eberl G, Sawa S (2010) Opening the crypt: current facts and hypotheses on the function of cryptopatches. Trends in Immunology 31: 50–55 10.1016/j.it.2009.11.004 20015688

[18] Glaysher BR, Mabbott NA (2007) Isolated lymphoid follicle maturation induces the development of follicular dendritic cells. Immunology 120: 336–344 10.1111/j.1365-2567.2006.02508.x 17163957 PMC2265896

[19] Knoop KA, Butler BR, Kumar N, Newberry RD, Williams IR (2011) Distinct Developmental Requirements for Isolated Lymphoid Follicle Formation in the Small and Large Intestine. The American Journal of Pathology 179: 1861–1871 10.1016/j.ajpath.2011.06.004 21854748 PMC3181393

[20] Hooper LV (2011) You AhR What You Eat: Linking Diet and Immunity. Cell 147: 489–491 10.1016/j.cell.2011.10.004 22036556

[21] Kiss EA, Diefenbach A (2012) Role of the Aryl Hydrocarbon Receptor in Controlling Maintenance and Functional Programs of RORγt(+) Innate Lymphoid Cells and Intraepithelial Lymphocytes. Front Immunol 3: 124 10.3389/fimmu.2012.00124 22666222 PMC3364460

[22] Bouskra D, Brézillon C, Bérard M, Werts C, Varona R, et al (2008) Lymphoid tissue genesis induced by commensals through NOD1 regulates intestinal homeostasis. Nature 456: 507–510 10.1038/nature07450 18987631

[23] Velaga S, Herbrand H, Friedrichsen M, Jiong T, Dorsch M, et al (2009) Chemokine Receptor CXCR5 Supports Solitary Intestinal Lymphoid Tissue Formation, B Cell Homing, and Induction of Intestinal IgA Responses. The Journal of Immunology 182: 2610–2619 10.4049/jimmunol.0801141 19234155

[24] Marchesi F, Martin AP, Thirunarayanan N, Devany E, Mayer L, et al (2009) CXCL13 expression in the gut promotes accumulation of IL-22-producing lymphoid tissue-inducer cells, and formation of isolated lymphoid follicles. Mucosal Immunol 2: 486–494 10.1038/mi.2009.113 19741597

[25] McDonald KG, McDonough JS, Dieckgraefe BK, Newberry RD (2010) Dendritic Cells Produce CXCL13 and Participate in the Development of Murine Small Intestine Lymphoid Tissues. The American Journal of Pathology 176: 2367–2377 10.2353/ajpath.2010.090723 20304952 PMC2861101

[26] Baptista AP, Olivier BJ, Goverse G, Greuter M, Knippenberg M, et al (2012) Colonic patch and colonic SILT development are independent and differentially regulated events. Mucosal Immunol 6: 511–521 10.1038/mi.2012.90 22990625 PMC3570605

[27] Williams IR (2006) CCR6 and CCL20: Partners in Intestinal Immunity and Lymphorganogenesis. Annals of the New York Academy of Sciences 1072: 52–61 10.1196/annals.1326.036 17057190

[28] Kuss SK, Best GT, Etheredge CA, Pruijssers AJ, Frierson JM, et al (2011) Intestinal Microbiota Promote Enteric Virus Replication and Systemic Pathogenesis. Science 334: 249–252 10.1126/science.1211057 21998395 PMC3222156

[29] Poltorak A, He X, Smirnova I, Liu MY, Van Huffel C, et al (1998) Defective LPS signaling in C3H/HeJ and C57BL/10ScCr mice: mutations in Tlr4 gene. Science 282: 2085–2088. 9851930 10.1126/science.282.5396.2085

[30] Akira S, Hoshino K (2003) Myeloid differentiation factor 88-dependent and -independent pathways in toll-like receptor signaling. J INFECT DIS 187 Suppl 2S356–S363 10.1086/374749 12792852

[31] Chen GY, Nuñez G (2009) Gut Immunity: A NOD to the Commensals. Current Biology 19: R171–R174 10.1016/j.cub.2008.12.027 19243695

[32] Shaw MH, Reimer T, Kim Y-G, Nuñez G (2008) NOD-like receptors (NLRs): bona fide intracellular microbial sensors. Current Opinion in Immunology 20: 377–382 10.1016/j.coi.2008.06.001 18585455 PMC2572576

[33] Kiss EA, Vonarbourg C, Kopfmann S, Hobeika E, Finke D, et al (2011) Natural Aryl Hydrocarbon Receptor Ligands Control Organogenesis of Intestinal Lymphoid Follicles. Science 334: 1561–1565 10.1126/science.1214914 22033518

[34] Bonizzi G, Karin M (2004) The two NF-kappaB activation pathways and their role in innate and adaptive immunity. Trends in Immunology 25: 280–288 10.1016/j.it.2004.03.008 15145317

[35] Williams A, Flavell RA, Eisenbarth SC (2010) The role of NOD-like Receptors in shaping adaptive immunity. Current Opinion in Immunology 22: 34–40 10.1016/j.coi.2010.01.004 20149616 PMC7038629

[36] Hardy ME, Hendricks JM, Paulson JM, Faunce NR (2012) 18β-glycyrrhetinic acid inhibits rotavirus replication in culture. Virol J 9: 96 10.1186/1743-422X-9-96 22616823 PMC3478227

[37] Robertson SJ, Zhou JY, Geddes K, Rubino SJ, Cho JH, et al (2013) Nod1 and Nod2 signaling does not alter the composition of intestinal bacterial communities at homeostasis. gutmicrobes 4: 222–231 10.4161/gmic.24373 PMC3669167 23549220

[38] Pan Q, Kravchenko V, Katz A, Huang S, Ii M, et al (2006) NF-B-Inducing Kinase Regulates Selected Gene Expression in the Nod2 Signaling Pathway. Infection and Immunity 74: 2121–2127 10.1128/IAI.74.4.2121-2127.2006 16552041 PMC1418900

[39] Hua H, Liang Z, Li W, Meng Y, Li X, et al (2012) Phenotypic and functional maturation of murine dendritic cells (DCs) induced by purified Glycyrrhizin (GL). International Immunopharmacology 12: 518–525 10.1016/j.intimp.2012.01.006 22293534

[40] Bordbar N, Karimi MH, Amirghofran Z (2012) Cellular Immunology. 280: 44–49 10.1016/j.cellimm.2012.11.013 23261828

[41] Biswas A, Petnicki-Ocwieja T, Kobayashi KS (2012) Nod2: a key regulator linking microbiota to intestinal mucosal immunity. J Mol Med 90: 15–24 10.1007/s00109-011-0802-y 21861185 PMC3263373

[42] Soulier A, Blois SM, Sivakumaran S, Fallah-Arani F, Henderson S, et al (2013) Cell-intrinsic regulation of murine dendritic cell function and survival by prereceptor amplification of glucocorticoid. Blood 122: 3288–3297 10.1182/blood-2013-03-489138 24081658

